# Effect of Acute Melatonin Injection on Metabolomic and Testicular Artery Hemodynamic Changes and Circulating Hormones in Shiba Goats under Sub-Tropical Environmental Conditions

**DOI:** 10.3390/ani13111794

**Published:** 2023-05-29

**Authors:** Haney Samir, Ahmed S. Mandour, Faten Radwan, Ahmed Ezzat Ahmed, Maha Abdullah Momenah, Nouf Arkan Aldawood, Tomihiko Yoshida, Gen Watanabe, Hossam R. El-Sherbiny

**Affiliations:** 1 Department of Theriogenology, Faculty of Veterinary Medicine, Cairo University, Giza 12211, Egypt; 2 Laboratory of Veterinary Physiology, Department of Veterinary Medicine, Faculty of Agriculture, Tokyo University of Agriculture and Technology, 3-5-8 Saiwai-Cho, Fuchu, Tokyo 183-8509, Japan; fatenradwan230@yahoo.com (F.R.); gen@cc.tuat.ac.jp (G.W.); 3 Department of Animal Medicine (Internal Medicine), Faculty of Veterinary Medicine, Suez Canal University, Ismailia 41522, Egypt; dr_mandour@vet.suez.edu.eg; 4 Laboratory of Veterinary Surgery, Tokyo University of Agriculture and Technology, Tokyo 183-8509, Japan; 5 Faculty of Veterinary Medicine, Benha University, Moshtohor, Toukh, Elqaliobiya 13736, Egypt; 6 Biology Department, College of Science, King Khalid University, Abha 61413, Saudi Arabia; ahmed.ezzat@vet.svu.edu.eg; 7 Theriogenology Department, Faculty of Veterinary Medicine, South Valley University, Qena 83523, Egypt; 8 Department of Biology, College of Science, Princess Nourah Bint Abdulrahman University, P.O. Box 84428, Riyadh 11671, Saudi Arabianoaraldawood@pnu.edu.sa (N.A.A.); 9 Division of Veterinary Research, Department of Veterinary Surgery, Obihiro University of Agriculture and Veterinary Medicine, Obihiro 080-8555, Japan; tomohiko7731-yoshida@yahoo.co.jp

**Keywords:** color Doppler ultrasonography, goats, hormones, heat stress, metabolomes, testicular artery blood flow

## Abstract

**Simple Summary:**

Due to climatic changes and global warming, heat stress has become a worldwide problem, especially in tropical and subtropical countries. Therefore, research studies that aim to alleviate heat stress conditions in males are valuable due to the high sensitivity of the testis to increased temperature. This study investigated whether an exogenous intravenous injection of melatonin could influence the testicular artery blood flow (TBF) and peripheral reproductive hormones and metabolomes in heat-stressed goats or not. Melatonin administration enhanced TBF, as measured by color Doppler ultrasonography, and induced significant changes in most studied hormones. Such data are valuable in the field of improving animal productivity under heat-stress environments because there were close correlations between TBF and potential animal fertility. In addition, peripheral metabolomic analysis was characterized and differentiated among the studied groups denoting several significant up and down metabolites that could be used in further research to investigate the underlying mechanisms.

**Abstract:**

The beneficial effects of melatonin were investigated to mitigate various detrimental effects and toxicity on reproductive performance. The present study aimed, for the first time, to explore the effect of intravenous melatonin injection on testicular artery hemodynamics (TH) and metabolomic changes, reproductive hormones in heat-stressed bucks. Ten bucks were randomly split into two groups (five each): (1) the melatonin group, treated with a single intravenous dose of melatonin solution containing 10 mg melatonin each, and (2) the control group, which was treated with 10 mL of the vehicle without melatonin. Changes in the TH at the level of the supra testicular artery (STA) were assessed by triplex ultrasonography just before (0 h) and at 0.5, 2, 7, 24, and 168 h after melatonin or vehicle administration. Doppler velocity parameters of peak systolic velocity (PSV; cm/s), end-diastolic velocity (EDV; cm/s), and time average maximum velocity (TAMAX; cm/s) were measured. Doppler indices (resistive index; RI and pulsatility index; PI), systole/diastole (S/D) ratio and total arterial blood flow volume (TABFV; ml/minute) were measured. Peripheral concentrations of FSH, LH, inhibin, melatonin, testosterone (T), estradiol (E2), and cortisol were measured just before injection (0 h) and at 0.5, 2, 7, and 24 h and daily up to day 7 post administration in both groups. Results revealed reductions in the RI values and increases in the TABFV in the melatonin group compared to the control one, especially 2 h after administration. Significant increases in concentrations of FSH, T, E2, and melatonin and decreases in cortisol and inhibin in the melatonin group compared to the control one. Plasma metabolomic analysis at 2 h indicated the up-regulation of L-glutamine, L-arginine, sorbitol, D-glucose, ascorbic acid, and ornithine and the down-regulation of D-xylose, D-arabitol, ribitol, and oleic acid in the melatonin versus the control group. In conclusion, acute administration of melatonin (10 mg IV) enhanced testicular artery blood flow and plasma reproductive hormones in the Shiba goat under heat-stress circumstances.

## 1. Introduction

Due to climatic changes and global warming, heat stress has become a worldwide problem and has gained more attention, especially in tropical and subtropical countries. Heat stress has substantial economic effects on animal performance and costs the global animal industry millions of dollars every year due to poor animal reproductive performances [1,2]. The effects of heat stress on reproduction are more profound because the affected animals showed reduced fertility for several weeks after the onset of thermal stress as a result of the response of the hypothalamic-hypophyseal-gonadal axis. In males, spermatogenesis and normal testicular function are temperature sensitive. For that reason, the testes in most mammals are suspended in the scrotum and outside the body to keep the testicular temperature of approximately 4–6 °C below the core body temperature [3]. The testes’ functions operate in a microenvironment close to hypoxia [4]. Therefore, increased ambient temperature in summer could negatively alter the testicular blood perfusion in goats [5], which has a pivotal role in regulating testicular temperatures [6,7]. Moreover, increases in the testicular temperature result in increases in metabolism and oxygen demand, hypoxia, production of reactive oxygen species (ROS) of the seminiferous tubules, and impairment of semen quality [8,9,10]. Therefore, research studies that aim to alleviate heat stress conditions are valuable.

Melatonin, a neurohormone of the pineal gland, plays a great role in attenuating oxidative stress conditions through its potency as a direct scavenger of free ROS [11]. In seasonal breeds of sheep and goats, melatonin was applied in the form of a slow-release implant to mimic the physiological rhythm of the short-day light/night cycle and regulate the testicular function and overall reproductive performance in males [12,13,14]. Previous experimental studies have revealed the beneficial effects of acute melatonin administration (through an intravenous injection) to mitigate various detrimental effects and toxicity that are induced by elevated ROS and oxidative stress conditions [11,15,16,17]. Melatonin (5 mg/kg) was given to rats intravenously 90 min after a transient focal cerebral ischemia; the findings demonstrate melatonin’s inhibitory ability against the cellular inflammatory response after cerebral ischemia-reperfusion [18]. Acute administration of melatonin as a potent ROS scavenger could be valuable in preventing and combating reproductive damage elicited by either continuous or intermittent hypoxia in mice [19]. Additionally, intravenous administration of melatonin protected the testes against oxidative stress damage caused by spinal cord injury in mice [20], as well as combating the acute ethanol-induced neurotoxic effects (such as elevated ROS, neuroinflammation, and neurodegeneration) in the developing rodent brain [21]. However, these experimental studies were mainly focused on laboratory animals, and a paucity of knowledge exists regarding the effects of intravenous administration of melatonin on testicular artery blood flow in multiple gastric animals, such as goats, as a model of ruminants, especially in the summer tropical season. Importantly, in severe circumstances of heat stress exposure, intravenous administration of a strong antioxidant is crucial to combat the undesirable effect of heat stress on testicular function. In recent years, metabolomic assessment has had a great impact and overreaching goal for its diagnostic and prognostic potentials, and their contributions to various biological phenomena have been popularly documented. Metabolomics can provide a comprehensive overview of the mechanism by measuring the full profile of small molecule metabolites in biological samples such as saliva, blood, and urine. Thus, it could help deepen our knowledge of metabolic pathways and their signatures relevant to various animal reproductive potentials [22]. To date, there are no studies investigating the effect of acute injection of melatonin through an intravenous infusion shot on metabolomic changes, reproductive hormones as well as testicular artery hemodynamics in goats. So, the purpose of this study was to investigate whether an exogenous intravenous injection of melatonin could influence or modulate the testicular artery blood flow and peripheral reproductive hormones and metabolomes in heat-stressed goats or not.

## 2. Materials and Methods

The present study was performed on male Shiba goats *(Capra hircus)* following the ethical guidelines of the local committee of Tokyo University of Agriculture and Technology, Japan, for the use of animals in experiments (Tokyo Nokodai, Fuchu, Tokyo, Japan; # 30–78).

### 2.1. Animals and Management

Ten sexually mature male Shiba goats (*Capra hircus*), 33.39 ± 2.85 months of age, weighing 36.90 ± 3.50 kg, and housed in a paddock under natural daylight conditions were used in the current study. They were fed a diet of 1200 gm of hay cubes per animal a day. Clean tap water and mineralized salt licks were available ad libitum. The goat paddock used belongs to the Laboratory of Veterinary Reproductive Physiology, Veterinary Medicine Department, Tokyo University of Agriculture and Technology, Japan (35.6690° N, 139.4777° E). All goats were free from any evidence of disease before the study and did not show any disorder during or after the experimental procedures. The animals were clinically healthy with good libido before the experiment. Moreover, each goat was exposed to a general examination, including the testis and epididymis, by ultrasonography to verify the absence of abnormalities in the reproductive tract before the study. Following the Japan Meteorological Agency, the average climatic data regarding the experimental period were 31.8 ± 0.8 °C, 81.4 ± 2.5%, and 86.4 ± 1.7 for temperature (T), relative humidity (RH), and temperature humidity index (THI), respectively. Bucks in this study were considered heat-stressed based on a previously published formula [23,24]: THI = (1.8 × T + 32) − [(0.55 − 0.0055 × RH) × (1.8 × T − 26)]. In which the bucks were categorized as non-stressed (THI < 70), moderately stressed (THI = 70–80), and severely stressed (THI > 80). Therefore, the examined bucks (THI = 86.4 ± 1.7) were considered severely heat-stressed bucks.

### 2.2. Experimental Design

**Figure 1:**
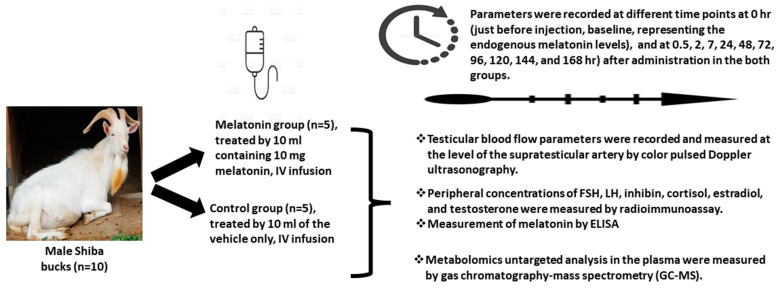
Schematic diagram of the experimental study.

The experimental design is presented in Figure 1. Goats were randomly split into 2 groups (5 goats each): (1) the Melatonin group, treated with a single intravenous dose of melatonin solution (10 mg each), and (2) the control group, which was treated with 10 mL of the vehicle without melatonin. Each buck was scanned using Triplex ultrasonography to assess the changes in testicular artery hemodynamics in the supra testicular artery (STA) at different time points [just before injection (0 h) and at 0.5, 2, 7, 24, and 168 h post-administration in both groups]. Concomitantly, blood samples were drawn [just before injection (0 h), at 0.5, 2, 7, 24 h, and daily (at 9.00 AM) up to day 7 post administration in both groups] to investigate the changes in the circulating metabolome and hormones.

#### How to Prepare and Inject Melatonin Intravenously?

On the day of the study, the solution of melatonin was prepared by mixing 2.5 mL ethanol (99.9%) with 50 mg of crystalline melatonin powder (Fujifilm Wako Pure Chemical Corporation, Osaka, Japan). Another 47.5 mL of saline 0.9% was added. This solution (1 mg melatonin/mL) was administered as a bolus (10 mL containing 10 mg melatonin) through a venous line at an infusion rate of 2.5 mL/minute over 4 min. The dose of melatonin, method of preparation, and intravenous administration of melatonin solution have been selected based on previously published studies and the pharmacokinetics of the exogenous intravenous (IV) administration of melatonin [25,26,27]. As previously indicated [28], the 0 h was selected to be at 09.00 h because it was considered a functional dead zone for melatonin action (melatonin administration at this time would not influence the circadian secretion of melatonin and would not be able to stop its reproduction. A previously published work on humans found a safe way of injecting very large doses of melatonin (up to 100 mg intravenously in adults) without adverse reactions [25].

### 2.3. Triplex Ultrasonographic Examinations

**Figure 2:**
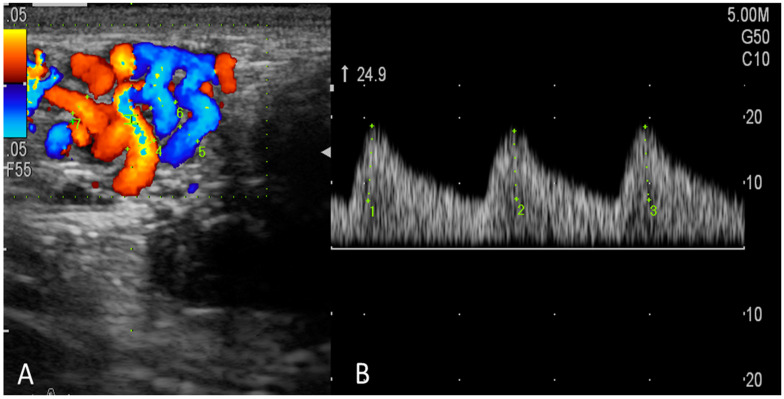
Imaging the testicular artery (at the supratesticular region, STA) by color Doppler ultrasonography (**A**) and the blood flow within the STA appeared as a spectral wave pattern of the testicular artery blood flow with a wave-like display (**B**).

Testicular artery blood flow (TBF) was monitored by the same operator (the first author) using a triplex ultrasound scanner (a ProSound F75 premier CV ultrasonography system; Hitachi Aloka Medical, Tokyo, Japan) that was equipped with an abdominal linear multi-frequency array transducer [6–14 MHz; probe (LN5415)]. As previously described [5,29], the bucks were simply secured, the hairs on both sides of the scrotum were removed via shaving, and a copious amount of ultrasonic gel was applied to the transducer to facilitate the assessment by ultrasonography. In the present study, we monitored testicular artery hemodynamics in supra testicular arteries (STA). After the vascular structures were identified and the largest longitudinal or oblique section of the STA was observed using B-mode ultrasonography, an assessment of TBF was carried out by color-pulsed Doppler ultrasonography as described previously [29,30] in goats. After the appearance of the spectral pattern of the STA (Figure 2), appended parameters were assessed: peak systolic velocity (PSV, cm/sec), end-diastolic velocity (EDV, cm/sec), and the time-averaged maximum velocity (TAMAX, cm/sec). Doppler indices studied were the resistive index (RI = (PSV − EDV)/PSV) and pulsatility index (PI = (PSV − EDV)/time average flow velocity). In addition, total arterial blood flow volume (TABFV) was measured using this equation [31]: TABFV = Cross-sectional area of the examined artery (A) × time-averaged velocity (TAV). In which A = D^2^ × 0.785; D is the radius (half of the diameter) of the examined artery. The diameter can be measured using the electronic caliber of B-mode ultrasonography. All spectral-Doppler settings (gains, focus, brightness, and contrast) were fixed and standardized. The angle between the long axis of the examined vessel and the Doppler beam was less than 60 degrees in the direction of blood flow, and the depth used ranged from 0.6 to 0.8 cm. The high-pass filter and the Doppler gate were set constant at 50 Hz and 1.5 m, respectively.

### 2.4. Blood Sampling and Hormonal Analysis

Blood samples were withdrawn from the jugular vein into heparinized tubes (Venoject II, Terumo, Tokyo, Japan) just before the injection (0 h: baseline that represents the endogenous levels of melatonin) and at 0.5, 2, 7, and 24 h interval up to one week after IV administration of the melatonin or the vehicle to goats. Before each sample was collected, about 2 mL of drawn blood from the catheter was discarded. Blood samples were then centrifuged at 3200 rpm (600× *g*) for 15 min at 4 °C; the plasma was separated, aliquoted, and stored at −20 °C until the assessment of concentrations of circulating hormones and metabolomes.

Plasma concentrations of FSH, LH, and inhibin were determined by a double-antibody radioimmunoassay (RIA) system using ^125^I- labeled radioligands as described in previous studies [32,33,34]. Plasma concentrations of FSH (ng/mL) were measured using anti-ovine FSH, NIDDK-oFSH-RP-1 as a standard reference, and NIDDK-FSH-I-1 for radio-iodination, while plasma LH (ng/mL) was measured using anti-ovine LH (YM 18), NIDDK-oLH-RP-24 as a reference standard, and NIDDK-oLH-I-3 for radio-iodination. Plasma concentration of inhibin (ng/mL) was measured using anti-bovine antiserum (TNDH-1) and bovine 32-kDa inhibin for radio-iodination. Concentrations of testosterone (T, ng/mL) and estradiol (E2, pg/mL) were measured in plasma as described by [35] using antisera against T (GDN 250) and E2 (GDN 244), respectively. Cortisol concentrations were measured [36] using radioiodination by rabbit anti-cortisol (HAC-AA71-02RBP85) and Cortisol-3-CMOBSA (80-IC20), and a reference standard of Hydrocortisone (H-4001). The intra- and inter-assay coefficients of variation were 9.6 and 11.8% for FSH, 5.6 and 6.8% for LH, 4.2% and 12.3% for inhibin, 8.4 and 9.6% for T, 5.6 and 7.7% for E2, and 2.4 and 9.4% for cortisol, respectively. Concentrations of melatonin (pg/mL) in the plasma were measured using a commercial ELISA kit (Biovision incorporated Co., Milpitas, CA, USA) and following the procedures of the enclosed instructions note. The absorbance values for the melatonin were read in a microtiter plate reader at 450 nm. The sensitivity of the melatonin ELISA kit is less than 4.7 pg/mL, the intra-assay CV is less than 8%, and the inter-assay CV is less than 10%. All hormonal analyses were carried out in triplicate and run in the same laboratory at Tokyo Nokodai, Fuchu, Tokyo, Japan.

### 2.5. Metabolomic Analysis

Untargeted metabolomics analysis of plasma samples was performed using Gas chromatography–mass spectrometry (GC/MS) as previously described [37,38,39] with little modifications. In brief, 50 μL of plasma sample was mixed with 5 μL of 2-isopropylmalic acid (1 mg/mL; Sigma-Aldrich, St. Louis, MO, USA) in distilled water as an internal standard. Afterward, a volume of 250 μL of methanol–chloroform–water mixture (2.5:1:1) was added, and the samples were subsequently mixed in a shaker at 1200 rpm at 37 °C for 30 min and then centrifuged at 16,000× *g* at for 5 min at 4 °C. Subsequently, a volume of 160 μL of the supernatant was mixed with 200 μL of distilled water and vortexed. Then, 250 μL of the supernatant was freeze-dried under a vacuum using a centrifugal evaporator (RD-400, Yamato Scientific Co., Ltd., Koto, Tokyo, Japan). Dried samples were then used after preparation for analysis of metabolites using the GC-MS machine. Then, a volume of 1 mL of pyridine dehydrate was used to dissolve 20 mg of methoxyamine hydrochloride (Sigma-Aldrich) was dissolved in pyridine for oximation. Then, a volume of 40 μL of this mixture was added to each dried sample, mixed well, and then shaken for about 90 min at 30 °C. Next, 20 μL of N-methyl N-trimethylsilyl-trifluoroacetamide (GL Science, Tokyo, Japan) was added for trimethylsilylation, and the mixture was incubated at 37 °C for 45 min. The samples were then subjected to the GC/MS (GCMS QP2010-Ultra; Shimadzu, Kyoto, Japan). The Shimadzu Smart Metabolites Database (Shimadzu, Kyoto, Japan) was used to identify metabolites. Samples were normalized by a pooling of all samples. The metabolomic analysis between two groups at the prominent time (2 h) was performed using MetaboAnalyst 5 as previously established [38,39]. The significant list of metabolites between both groups was subjected to an enrichment analysis.

### 2.6. Statistical Analysis

The GraphPad prism5 software was used for all statistical analyses. Data were exposed to a normality test using the Kolmogorov–Smirnov test in GraphPad Prism to identify the homogeneity and the type of data. In this study, the studied hemodynamic and echotextural parameters showed no significant differences between the right and left testes; thus, the data for each buck was pooled and comparisons were carried out among the two groups. Data for Doppler parameters of the testicular artery hemodynamic at the STA and the hormonal results were presented as means ± standard error of the mean (SEM). Means were analyzed for the difference using repeated measures two-way ANOVA to study the effect of the treatment as a fixed factor and the time as a repeated factor. The effect of treatment (2 levels; acute melatonin injection versus the control) on the aforementioned studied parameters was tested along different time points, followed by the Bonferroni post hoc test. A value of *p* < 0.05 was considered significant.

## 3. Results

### 3.1. Testicular Artery Hemodynamics

**Figure 3:**
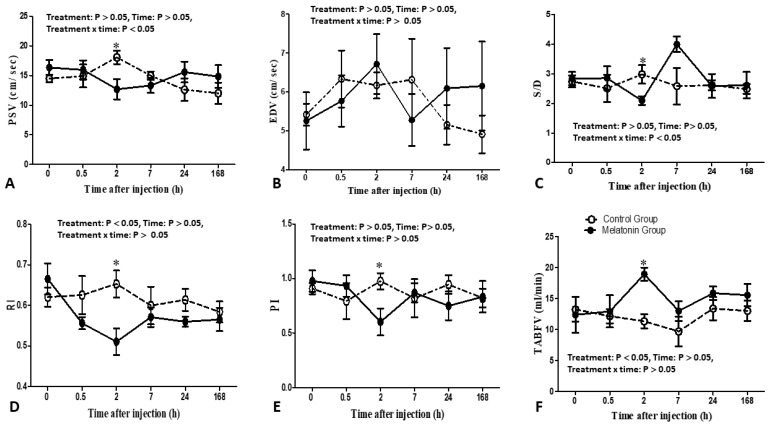
Changes in the measured parameters of testicular artery blood flow [peak systolic velocity, (PSV, cm/sec; (**A**)); end-diastolic velocity (EDV, cm/sec; (**B**)); systolic velocity: end-diastolic velocity ratio (S/D; (**C**)); resistive index (RI; (**D**)); pulsatility index (PI; (**E**)); and total arterial blood flow volume (TABFV, mL/min; (**F**))] as assessed by color pulsed Doppler ultrasonography in Shiba bucks that received either a single intravenous administration of melatonin (closed circles; melatonin group; *n* = 5) or the vehicle (open circles; control group; *n* = 5). ∗ Values differed significantly from the corresponding values in the control group at the specific time points.

The effect of melatonin injection on testicular artery hemodynamics is presented in Figure 3. In general, the effect of treatment (two levels: melatonin group versus control group) and interaction had significant effects on the RI values and TABFV at the examined STA (*p* < 0.05). Bucks in the melatonin group had lower RI values (*p* < 0.05) compared to the control bucks, and the RI values were lesser, especially at 2 h after intravenous administration of melatonin (0.510 ± 0.032) compared to its values in the control group (0.653 ± 0.033) (*p* < 0.05). Significant increases in the TABFV (mL/min) were found in the melatonin-treated bucks compared to that in the control one (*p* < 0.05). The TABFV was higher at 2 h (18.94 ± 1.06 mL/min) after administration of melatonin compared to its corresponding values (11.33 ± 1.13 mL/min) in the control group (*p* < 0.05). However, treatment, time, and the interaction factor (treatment x time) had no significant effects on other parameters of the testicular artery hemodynamic at the level of STA. Notable decreases in values of PI, PSV, and S/D Ratio (*p* > 0.005) were observed in TBF in the melatonin group compared to the control group at 2 h after injection.

### 3.2. Circulating Hormones

**Figure 4:**
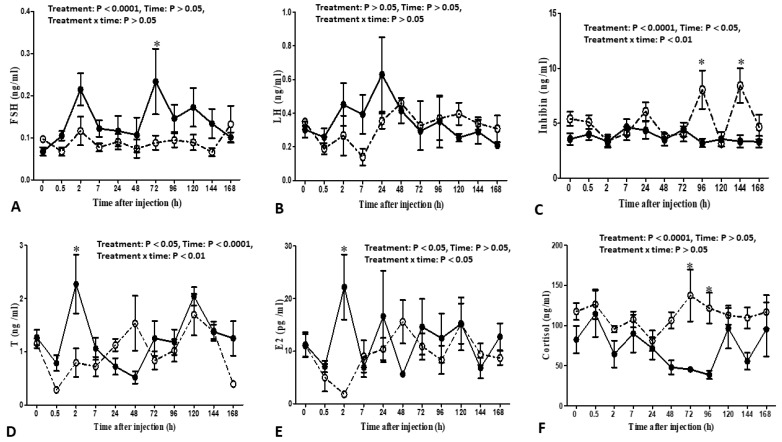
Changes in the plasma concentrations of follicle-stimulating hormones (FSH, ng/mL, (**A**)); luteinizing hormone (LH, ng/mL, (**B**)); inhibin (ng/mL, (**C**)); testosterone (T, ng/mL, (**D**)); estradiol (E2 pg/mL, (**E**)); and cortisol (ng/mL, (**F**)) as measured by radioimmunoassay in Shiba bucks that received either a single intravenous administration of melatonin (closed circles; melatonin group; *n* = 5) or the vehicle (open circles; control group; *n* = 5). ∗ Values differed significantly from the corresponding values in the control group at the specific time points.

**Figure 5:**
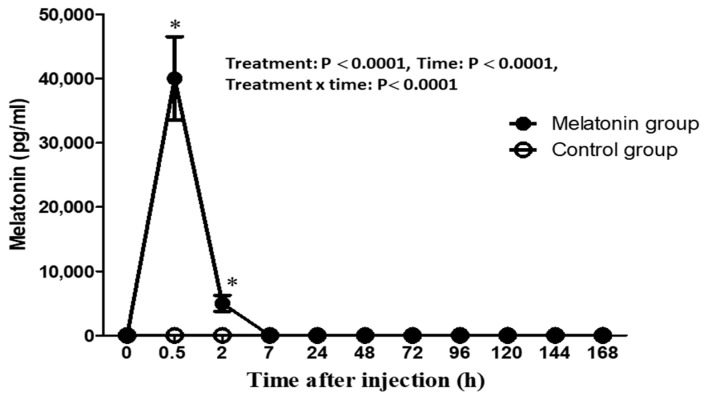
Changes in the plasma concentrations of melatonin (pg/mL) as measured by ELISA in Shiba bucks that received either a single intravenous administration of melatonin (closed circles; melatonin group; *n* = 5) or the vehicle (open circles; control group; *n* = 5). ∗ Values differed significantly from the corresponding values in the control group at the specific time points.

The effect of intravenous administration of melatonin bolus on reproductive hormones is presented in Figure 4 and Figure 5. In general, the effect of administration (melatonin versus control) had significant effects on the concentrations of circulating cortisol (*p* ˂ 0.0001), testosterone (*p* ˂ 0.05), estradiol (*p* ˂ 0.05), FSH (*p* ˂ 0.05), inhibin (*p* ˂ 0.0001), and melatonin (*p* ˂ 0.0001). The time factor had a significant effect on the concentrations of circulating testosterone (*p* ˂ 0.0001), inhibin (*p* ˂ 0.05), and melatonin (*p* ˂ 0.0001). The interaction factor showed a significant effect on the circulating cortisol (*p* ˂ 0.05), testosterone (*p* ˂ 0.001), estradiol (*p* ˂ 0.05), FSH (*p* ˂ 0.05), inhibin (*p* ˂ 0.001), and melatonin (*p* ˂ 0.0001). However, there were no treatment, time, and interaction effects on the concentrations of LH during the study. Bucks in the melatonin group had lower cortisol levels than those in the control group (*p* ˂ 0.0001). Lower cortisol levels were found in the melatonin group, especially at 72 h and 96 h compared to the control group (*p* ˂ 0.0001). High testosterone levels were observed in the melatonin group compared to those in the control group (*p* ˂ 0.0001). The overall means of testosterone in the melatonin group were significantly higher at 2 h (2.27 ± 0.56 ng/mL) versus 0 h (1.27 ± 0.14 ng/mL) and were significantly higher at 2–3 h after acute administration of melatonin (*p* ˂ 0.0001) versus the control group (0.79 ± 0.27 ng/mL). Increased levels of estradiol were found in the melatonin group compared to the control group, and its levels were obviously (*p* ˂ 0.05) higher at 2 h (22.18 ± 6.18 pg/mL) compared to the control one (1.83 ± 0.48 pg/mL). High levels of FSH were found in the melatonin-administered bucks compared to those in the control-treated bucks (*p* ˂ 0.05). High levels of FSH were noticed (*p* ˂ 0.05) at 72 h (0.23 ± 0.08 ng/mL) in the melatonin group versus the control one (0.09 ± 0.02 ng/mL). Conversely, decreased levels of inhibin were observed in the melatonin group compared to those in the control one, and the lowest concentrations were observed at 96 h (3.19 ± 0.40 ng/mL) and 144 h (3.33 ± 0.56 ng/mL) in the melatonin group versus its corresponding values in the control one (8.05 ± 1.75 ng/mL, 8.04 ± 1.57 ng/mL, respectively) (*p* ˂ 0.001). Bucks in the melatonin group had significant increases in the concentrations of melatonin compared to the control group (*p* ˂ 0.001). Melatonin concentrations showed robust increases (*p* ˂ 0.001) at 30 min (40000.67 ± 6500.77 pg/mL) after melatonin administration, robust decreased at 2 h (5000.32 ± 1250.95 pg/mL) and reached lower concentrations (similar to baseline levels) at 7 h (9.76 ± 2.31 pg/mL) after administration. However, its values in the control group showed non-significant changes (*p* > 0.001).

### 3.3. Metabolomic Changes

**Figure 6:**
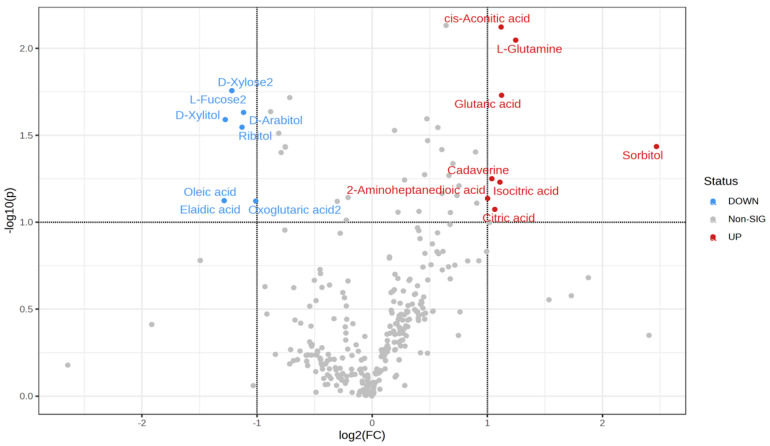
Volcano plots of the changes in the plasma metabolomic profiles of the melatonin and control group. The identification of significantly altered metabolites (upregulated, downregulated, and non-significant). Red circles indicate the up-significant metabolites while the blue ones indicate the down-significant metabolites in the melatonin group compared to the control group.

**Figure 7:**
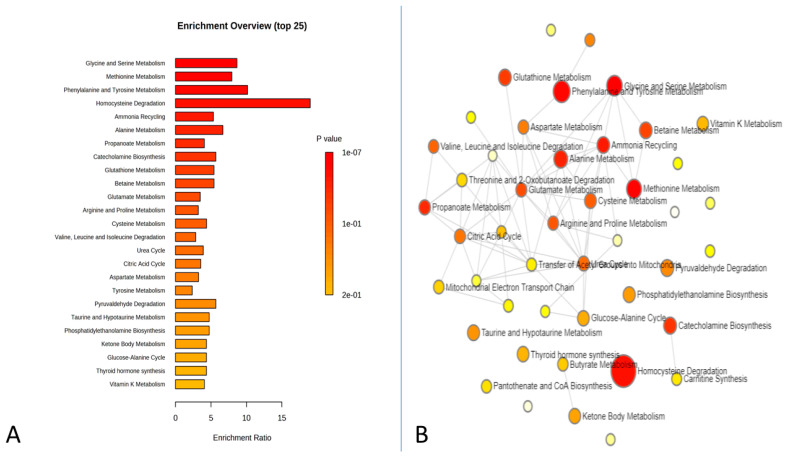
Enrichment analysis of the significant metabolites showed top pathways that may be involved in these changes. (**A**) Enrichment Overview (top 25); (**B**) Finding of network analysis.

**Figure 8:**
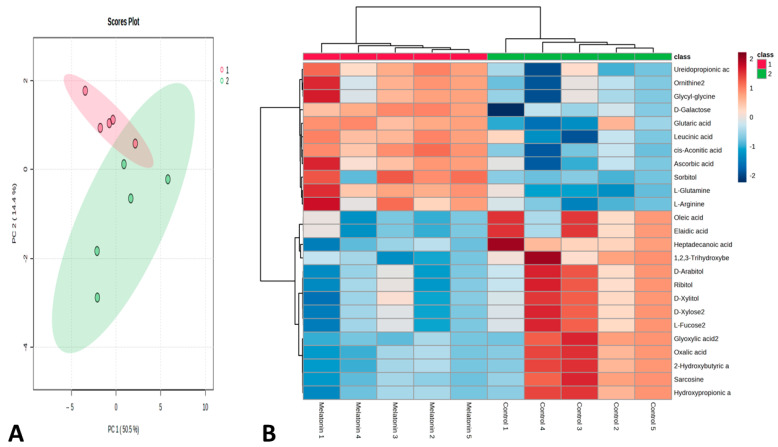
Differences in the metabolic components between the melatonin and control group (**A**). Heatmap analysis of the relative significant metabolites that were found between the two groups (*n* = 5 each) in the plasma of goats at 2 hours (a fold change threshold of 2 and a *t*-test threshold of *p* < 0.05) (**B**).

The effect of the administration of melatonin (an intravenous bolus) on metabolomic changes in the plasma at 2 h (the time characterized by high blood perfusion to the testis) is presented in Figure 6, Figure 7 and Figure 8. Results revealed an up- and down-regulated number of metabolites among the studied group. In this study, we performed an untargeted metabolomics analysis of the metabolite profiles of plasma for 2 h using the GC-MS. To further analyze the differences between the control and melatonin groups, a volcano plot was constructed to identify several significant metabolites with a fold change (FC) threshold of 2 and a t-test threshold of *p* < 0.05. Untargeted metabolomic analysis of the plasma samples detected 319 non-significant metabolites and 6 significant ones (8 metabolites were significantly upregulated and 8 ones were significantly downregulated) (Table S1). The histogram denotes the relative abundance of several metabolites (Figure 6). The enrichment analysis of the significant metabolites showed top pathways that may be involved in these changes, such as glycine and serine metabolism, methionine metabolism, phenylalanine, tyrosine metabolism, homocysteine degradation, ammonia recycling, and alanine metabolism (Figure 7). Differences in the metabolic components between the melatonin and control groups are presented in Figure 8A. A heat map of the relative significant metabolites that were found between the two groups is shown in Figure 8B.

## 4. Discussion

This study is the first that addressed the effect of acute administration of melatonin on TBF and various reproductive hormones in bucks under ambient heat stress conditions and along selected time points based on previously published reports and the pharmacokinetics of melatonin after intravenous administration [26]. The results of the current study indicated the positive effect of melatonin injections on the detrimental effects caused by heat stress conditions in male goats. Melatonin administration enhanced TBF as measured by color Doppler ultrasonography. Concomitantly, significant changes in most studied hormones were found in the melatonin-treated group. Such data are valuable in the field of improving animal productivity under heat-stress environments because there were close correlations between TBF and potential animal fertility [4,40]. In addition, peripheral metabolomic analysis was characterized and differentiated among the studied groups denoting several significant up and down metabolites that could be used in further research to investigate the underlying mechanisms. 

In the current study, acute administration of melatonin induced an enhanced testicular artery hemodynamic (induced a stimulatory effect on TBF) at the level of the STA in male goats, as evidenced by lower Doppler indices. Similar findings were reported after a single slow release of melatonin dose suspended in corn oil was administered S/C in Shiba goats and could potentially improve male goats’ fertility [41]. The exact mechanism of action of melatonin is unclear. It may be attributed to its potent antioxidant effects [42]. In vivo and in vitro studies demonstrated that acute melatonin administration acted as a potent endogenous antioxidant neuroprotective neurohormone that stimulated the master endogenous antioxidant Nrf2 and ameliorated the detrimental effects caused by acute ethanol-induced elevated ROS, neuroinflammation, and neurodegeneration in the developing rat brain and could be beneficial to prevent neurotoxicity in fetal alcohol syndrome [21]. Accordingly, bucks in the melatonin group had lower cortisol levels than those in the control group (*p* ˂ 0.0001).

In the present study, the improvement of TBF in the melatonin group might be attributable to its stimulatory action on cardiac contractility [43,44,45] rather than its direct effects on the testicular vasculature. However, the cardiac functions were not assessed in the present study to prove this explanation. The melatonin group had lower RI values (that indicated increases in TBF) compared to the control bucks, and the RI values were lesser, especially at 2 h. Additionally, E2 levels increased 2 h after the administration of melatonin, which may act as a strong vasodilator [41,46,47]. Melatonin has a crucial role in the conversion of androgen into estrogen by regulating aromatase transcription [48]. Considering the lipophilic nature of the hormone, interactions with specific intracellular proteins [49,50] or nuclear receptors cannot be excluded; however, melatonin seems to exert its effects principally throughout high-affinity G-protein-coupled receptors [51]. However, another potential effect of exogenous melatonin should be considered. A single application of melatonin within the SCN, in vivo, induced a long-lasting increase in the amplitude of the nocturnal melatonin secretion [52]. Moreover, it was demonstrated that exogenous melatonin could sustain the oscillation of the clock and suggests a possible role for endogenous melatonin in mammals [51].

In addition to its effect on TBF, levels of cortisol, and E2, other reproductive hormones changed significantly after the acute administration of melatonin. In the current study, bucks that received melatonin had increased levels of T and FSH. Increased levels of T in bucks that received an IV administration of melatonin were consistent with previous studies in goats [53] and rams [54,55]. Increased T may be due to the stimulatory effect of melatonin on some steroidogenic enzymes, such as 3beta-hydroxysteroid dehydrogenase, as previously reported [56]. Increased levels of FSH in the melatonin group may be attributable in part to the positive effect of melatonin administration on its secretion, as previously stated [57]. Conversely, lower levels of inhibin were found in the melatonin group compared to the control one. A previous study in rats indicated that melatonin could reduce the suppression effect (31%) of inhibin hormone on FSH levels as compared to the non-melatonin group (51%) [58].

Melatonin receptors were investigated in the cardiovascular systems. Therefore, the second explanation for increased TBF in the melatonin group could be attributable to the effect of melatonin on cardiac functions [44,59,60,61]. The findings from human and animal studies supported the consideration of melatonin as a cardioprotective agent [44]. Melatonin serves as an endocrine regulator of nighttime blood pressure in healthy adults by contributing to its normal decline during sleep by 10–20% from its daytime mean level [59]. In addition, melatonin acts as a potent radical scavenger, reducing oxidative stress caused by arterial hypertension, and it improves endothelial function by increasing nitric oxide bioavailability, which may reduce blood pressure through the attenuation of peripheral arterial resistance [60]. Melatonin also inhibits the vasospastic effect of hydrogen peroxide on the human umbilical artery [61]. Therefore, it has been suggested that melatonin, having several pleiotropic actions, might be protective of the cardiovascular system and also against the development of hypertension and organ damage. Reduced levels of nighttime circulating melatonin have been found in pregnant women with severe preeclampsia in comparison with women with normal pregnancies or with mild preeclampsia [62]. Dysfunction of the circadian system and secretion of endogenous melatonin results in dysregulation of fetal cardiac gene expression, increasing the risk of cardiovascular disorders in adult offspring [63]. Recently, Chaves et al. [64] showed that the offspring of mice exposed to repeated jet lag during pregnancy exhibit structural alterations of heart tissue and impaired heart function. Moreover, melatonin prevents cardiac hypertrophy in hyperthyroid rats, reduces oxidative load, and alters the expression of metabolically important genes [65].

In the present work, there were significant changes in the number of plasma metabolites between the two groups. Significant upregulation of some metabolites, such as l-glutamine, cis-aconitic acids, l-arginine, ureidopropionic acid, ascorbic acid, ornithine, and sorbitol, was found in the melatonin group compared to the control one. These findings of the plasma metabolomic components may refer to either the direct or indirect effects of melatonin on different biological pathways. Significant upregulation of l-glutamine may point to its regulatory roles on cardiac blood flow and fluidity by generating nitric oxide [66]. Additionally, increased l-arginine could be involved in increasing the synthesis and release of nitric oxide and the testicular artery blood flow, which improves the performance of the testis in the production of testosterone hormone, as previously reported [67]. Other metabolites are involved in both energy production and removal of toxic ammonia, such as cis-aconitic acids or various metabolic pathways (beta-alanine metabolism and pyrimidine metabolism), such as ureidopropionic acid. Sorbitol functions as a substrate in the polyol pathway where unused glucose is reduced into sorbitol, which in turn, is oxidized to produce fructose (an essential monosaccharide that is incorporated in certain metabolic activities of the cell, such as fructolysis and glycation). In addition, sorbitol has been suggested as a suitable biomarker to assess hepatic blood perfusion [68]. Upregulation of L-ascorbic acid in the melatonin administered bucks may explain the increases in TBF in this group. The previous literature indicated the significant protective effects of L-ascorbic acid against oxidative stress damage to the testis in rats [69]. However, to elucidate the mechanism in the present study, further studies may be imperatively needed in this regard.

## 5. Conclusions

Collectively, it can be concluded that acute administration of melatonin (10 mg IV) to male Shiba goats enhanced testicular artery blood flow as measured by color-pulsed Doppler ultrasonography through decreasing RI values of blood flow within the STA. Concomitantly, most peripheral hormones showed significant alterations (increases in levels of FSH, T, and E2, and decreases in the levels of cortisol and inhibin) observed in the melatonin-administered group. Melatonin concentrations decreased to their baseline levels within 7 h of administration. Peripheral metabolomic analysis was characterized and differentiated among the studied groups, denoting a number of significant up- and down metabolites. Further investigations will be valuable owing to illustrating the mechanism, at least at the testis level. This work was considered a preliminary study to enhance various reproductive parameters in bucks under high-temperature conditions. However, the study of cardiac parameters, semen quality, and fertility rate on a large number of animals may be needed in further studies.

## Data Availability

All data generated during this study are included in this published article and its Supplementary file.

## Supplementary Materials

The following supporting information can be downloaded at: https://www.mdpi.com/article/10.3390/ani13111794/s1, Table S1: Results of a volcano plot that was constructed to identify several significant metabolites with a fold change (FC) threshold of 2 and a *t*-test threshold of *p* < 0.05 in Shiba bucks received a single intravenous administration of melatonin compared to the control ones.
