# Supplementary material for: Effect of Acute Melatonin Injection on Metabolomic and Testicular Artery Hemodynamic Changes and Circulating Hormones in Shiba Goats under Sub-Tropical Environmental Conditions
Source: Animals (Basel). 2023 May 29;13(11):1794. doi: 10.3390/ani13111794 (PMC10252098; doi:10.3390/ani13111794)
Supplement: Supplementary file 1 [file animals-13-01794-s001.zip › animals-2322980-supplementary.pdf]

Table S1. Results of a volcano plot that was constructed to identify a number of significant metabolites with a fold change (FC) threshold of 2 and a t-test threshold of P < 0.05 in Shiba bucks received a single intravenous administration of melatonin compared to the control ones.

	**Metabolites**	**FC**	**log2(FC)**	**P-value**
	cis-Aconitic acid	2.1726	1.1194	0.0075409
	L-Glutamine	2.3717	1.2459	0.0089712
	Glutaric acid	2.1784	1.1233	0.018615
	Sorbitol	5.5344	2.4684	0.03672
	Cadaverine	2.0543	1.0387	0.056223
	Isocitric acid	2.1566	1.1087	0.058856
	2-Aminoheptanedioic acid	2.0028	1.002	0.073025
**Up**	Citric acid	2.0914	1.0645	0.084384
	D-Xylose2	0.42961	-1.2189	0.017512
	L-Fucose2	0.42961	-1.2189	0.017512
	D-Arabitol	0.46103	-1.1171	0.023376
	D-Xylitol	0.4129	-1.2762	0.025696
	Ribitol	0.45673	-1.1306	0.02842
	Oleic acid	0.41014	-1.2858	0.075205
	Elaidic acid	0.41014	-1.2858	0.075205
**Down**	Oxoglutaric acid2	0.49601	-1.0116	0.075616
